# Greasy hair against obesity

**DOI:** 10.1038/s41392-021-00850-7

**Published:** 2021-12-17

**Authors:** Sandra Nickel, Bruno Christ

**Affiliations:** 1 Department of General, Visceral and Vascular Surgery, Jena University Hospital, 07740 Jena, Germany; 2 Cell Transplantation/Molecular Hepatology Lab, Department of Visceral, Transplant, Thoracic and Vascular Surgery, University of Leipzig Medical Center, 04103 Leipzig, Germany

**Keywords:** Translational immunology, Molecular medicine

In their article, Choa et al. open the perspective to loose fat by increasing sebogenesis. Envisioning a T cell-dependent mechanism, they suggest that stimulation of sebum production by thymic stromal lymphopeietin (TSLP) “pulls” lipids out of the adipose tissue.^1^

**Fig. 1:**
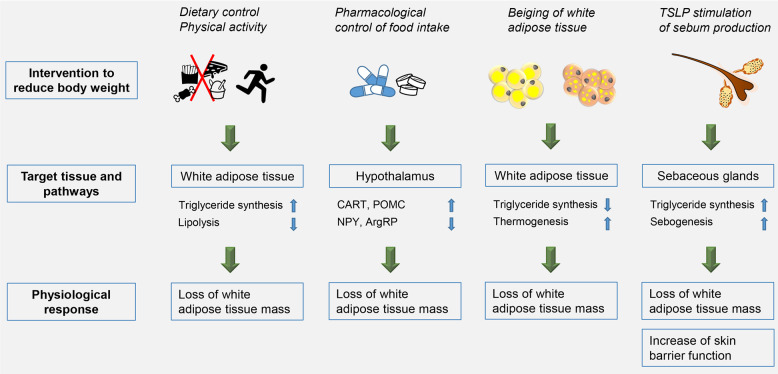
Strategies to reduce adipose. Restriction of calorie intake and promotion of physical activity directly require individual initiative and action, while pharmacological interventions passively aim to decrease food uptake, e.g., via orexigenic (NPY, neuropeptide Y; AgRP, agouti-related peptide) or anorexigenic (CART, cocaine- and amphetamine-related transcript; pro-opiomelanocortin, POMC) neuropeptides. Beiging of white adipose tissue by, e.g., cold stress addresses fat depot breakdown via thermogenesis. TSLP activation of sebum production “sucks” off lipids from adipose tissue, but in addition impacts on skin barrier homeostatic functions

The World Population Review (worldpopulationreview.com/) ranks obesity rates to one third of the global population. Associated co-morbidities comprise diabetes type 2, ischemic stroke and cardiovascular complications render this epidemic disease the number 1 cause of deaths worldwide (who.int/). Treating obesity encompasses besides classical approaches like dietary restrictions combined with increased physical activity more and more the targeting of molecular pathways involved in the pathogenesis and persistence of obesity. E.g., the discovery of “beige” adipocytes in white adipose tissue (WAT), which share lipid utilization via thermogenesis with “brown” adipocytes, fostered the hype to induce therapeutic “beiging” of WAT by cold stress and pharmacological interventions (Fig. 1).

Choa and colleagues suggest to eliminate fat via the skin by stimulating sebum excretion from sebocytes by TSLP.^1^ They discovered that overexpression of TSLP in ob/ob mice and in mice fed a high fat diet caused weight loss by decreasing visceral fat mass and improving metabolic homeostasis. In mouse models featuring lack of different immune cell sub-populations, they identified activated and/or memory T cells to mediate TSLP-induced WAT loss by a receptor-dependent, but the antigen-independent mechanism. WAT disappearance did not involve reduced calorie uptake, increased consumption by thermogenic expenditure or physical activity, and not excretion of high energy metabolites via the faeces. Overexpression of TSLP rather increased hair grease in consequence of augmented sebum production due to higher sebocyte turnover. While TSLP obviously stimulated lipolysis in the adipose tissue, neither free fatty acids nor triglycerides increased in the serum attributable to the absorption of serum lipids by sebocytes for their TSLP-stimulated sebum production. What then is the role of T cells in this scenario? T cells were necessary for TSLP receptor-mediated hair grease production; CD4^+^ and CD8^+^ cells were enriched in direct vicinity of sebaceous glands after TSLP stimulation; Inhibition of T cell migration abrogated TSLP-stimulated T cell enrichment in the skin, loss of WAT, and sebum over-production; The latter was achieved by receptor stimulation on T cells. Comparing wildtype with TLSP receptor knockout mice and with B and T cell-deficient mice revealed that the manipulated mice featured less hair wax esters, lower sebocyte turnover and less antimicrobial peptides in the sebum, which defines a role of immune cell-mediated TSLP regulation of skin barrier homeostasis. Finally, 18 genes in human sebaceous glands from healthy skin correlated with TSLP expression suggesting a role of TSLP in sebum regulation also in humans. In line with these findings, TSLP treatment ameliorated atherosclerosis in the ApoE knockout mouse model by a dendritic and regulatory T cell-mediated mechanism suggesting that TSLP might regulate cholesterol metabolism in addition to lipid metabolism in adipose tissue as shown in the study by Choa et al. This confirms a more general impact of TSLP on the involvement of immune cells in the regulation of lipid metabolism.^2^

While nutritional control by dietary or pharmacological interventions directly target the adipose tissue with the aim of fat tissue mass reduction, TSLP-stimulation of sebum production indirectly relates to the adipose tissue (Fig. 1). The regulation of sebum production by sebaceous glands interferes on multiple levels with the regulation of food intake and the storage and mobilization of energy substrates from the organs involved like adipose tissue, liver, and muscle. Besides regulation by TSLP, sebogenesis is under extensive neurohormonal control along the hypothalamus-pituitary route in the context of adrenal, thyroid, growth, and sexual hormones, as well as by the adaptive and innate immune systems. This reflects the manifold tasks of the skin in sexual and flight behavior, thermo- and water balance regulation, and skin immune barrier functions. In adipose tissue, TSLP expression is under the control of thyroid-stimulating hormone (TSH). Interestingly, expression was lower in obese men with metabolic syndrome compared with those without metabolic syndrome indicating an association of TSLP with whole-body energy homeostasis.^3^ The common regulation of TSLP expression in adipose tissue and sebum production in sebocytes by TSH may thus suggest a functional link between the regulation of body energy balance and utilization of adipose-derived lipids in sebum production by a feed-forward mechanism. Data shown by Choa et al.^1^ underscore this by showing that TSLP-induced lipid loss was not confined to the adipose tissue alone, but also engaged lipid breakdown in livers of mice suffering from non-alcoholic steatohepatitis (NASH).^1^ In the NASH livers, TSLP also reduced hepatocyte damage, which is the consequence of lipid-induced oxidative stress and inflammation. TSLP might directly affect hepatocytes, since it stimulated hepatocyte autophagy after ischemia/reperfusion injury (I/R) by activation of the PI3-kinase pathway. This reduced liver damage in the I/R-induced sterile inflammatory environment,^4^ a scenario, which is closely linked also to the pathogenesis of the metabolic syndrome.

Mouse and human TSLP are different. While in the mouse only one form exists, humans feature a long and a short isoform of TSLP. In mice, TSLP at physiological levels seems involved in homeostasis regulation, while overexpression supports adipose loss via increased sebum production. In humans, this relationship remains elusive. Current knowledge rather suggests that the long isoform is upregulated in Th2-mediated inflammatory allergic reactions like asthma and atopic dermatitis. The short form is constitutively expressed by, e.g., stimulation by commensal bacteria, and is involved in homeostatic regulation like maintenance of physiological gut barrier functions. The stimulation of epithelial cells by pathogens induces secretion of the long form.^5^ Yet, so far it is unclear, which form might be involved in sebocyte regulation in humans. Therefore, fat waste by TSLP-stimulated sebum production remains to be established unequivocally. Albeit the possibility to loose fat through the skin is intriguing, the stringent regulation of sebacious glands by hormones and neuropeptides and their feedback control requires careful evaluation before the clinical launch of TSLP as fat waist option. Hence, be aware of not casting out the Fiend with the devil of inflammatory skin and other organs´ diseases.
